# Inhibitor of DNA binding/differentiation 4 deficiency impairs hepatic fatty acid synthesis and is associated with epigenomic alterations in chromatin accessibility

**DOI:** 10.1016/j.molmet.2026.102416

**Published:** 2026-07-08

**Authors:** Yoshikazu Hayashi, Koji Kinoshita, Tsai-Ming Lu, Hsin-Yi Tseng, Keita Maki, Soi Kimura, Ena Yano, Ayaka Saeki, Atsushi Yasukochi, Kento Minami, Mayo Yamamura, Ichiro Takahashi, Masato Hirata, Eijiro Jimi, Lo Yi-Chen, Cheng-Fu Kao, Tomoyo Kawakubo-Yasukochi

**Affiliations:** 1 OBT Research Center, Faculty of Dental Science, Kyushu University, 3-1-1 Maidashi, Higashi-ku, Fukuoka 812-8582, Japan; 2 Division of Functional Structure, Department of Morphological Biology, Fukuoka Dental College, Tamura, Sawara-ku, Fukuoka 814-0193, Japan; 3 Oral Medical Research Center, Fukuoka Dental College, Tamura, Sawara-ku, Fukuoka 814-0193, Japan; 4 Section of Orthodontics and Dentofacial Orthopedics, Division of Oral Health, Growth and Development, Kyushu University Faculty of Dental Science, Fukuoka, Japan; 5 Institute of Cellular and Organismic Biology, Academia Sinica, Taipei, Taiwan; 6 Section of Oral and Maxillofacial Oncology, Division of Maxillofacial Diagnostic and Surgical Sciences, Faculty of Dental Sciences, Kyushu University, 3-1-1 Maidashi, Higashi-ku, Fukuoka 812-8582, Japan; 7 Laboratory of Molecular and Cellular Biochemistry, Faculty of Dental Science, Kyushu University, 3-1-1 Maidashi, Higashi-ku, Fukuoka 812-8582, Japan; 8 Dental Maxillofacial Center, Kyushu University Hospital, 3-1-1 Maidashi, Higashi-ku, Fukuoka 812-8582, Japan; 9 Institute of Food Sciences and Technology, National Taiwan University, Taipei 10617, Taiwan

**Keywords:** ID4, Fatty acid synthesis, SREBP1, ACC1, FASN, Chromatin accessibility

## Abstract

Inhibitor of DNA binding/differentiation (ID) 4 is a member of the ID family of proteins. ID4 is involved in gene transcriptional regulation during diverse pathophysiological processes, including cellular differentiation, proliferation, and senescence. ID4-deficient (*Id4*^*−/−*^) mice exhibit markedly reduced tissue and body mass and survive for only a few weeks after birth. However, the direct cause of this premature lethality remains unknown. This study demonstrated that ID4 deficiency leads to impaired hepatic fatty acid synthesis, accompanied by downregulation of the rate-limiting enzymes of fatty acid synthesis, such as fatty acid synthase (FASN) and acetyl-CoA carboxylase 1 (ACC1). Comprehensive histone modification profiling based on mass spectrometry revealed drastic alterations involving histone modification patterns in the livers of *Id4*^*−/−*^mice compared to those of wild-type littermates. Furthermore, ID4 deficiency resulted in aberrant histone expression patterns. Integrative assay for transposase-accessible chromatin sequencing and RNA-sequencing analyses further revealed that ID4 deficiency induces chromatin closure at the promoter region of *Srebf1* (encoding sterol regulatory element-binding protein), a master transcription factor upstream of *Fasn* (encoding FASN) and *Acaca* (encoding ACC1). Collectively, these findings indicate that ID4 functions as an important regulator of hepatic fatty acid metabolism by maintaining chromatin accessibility, rather than merely acting as a classical ID protein that regulates target gene transcription.

## Highlights

• *Id4*-deficient mice exhibit impaired hepatic fatty acid synthesis.
• *Id4*-deficient mice exhibit reduced FASN and ACC1 expression.
• *Id4*-deficient mice show changes in histone expression and modification in liver.
• Id4 maintains chromatin accessibility at *Srebf1* promoter.
• *Srebf1* promoter enables expression of transcription factor for *Fasn* and *Acc1*.

## 1 Introduction

Fatty acid metabolism is essential for maintaining cellular and systemic functions necessary for survival [1,2]. The process has also been proposed as a key regulator of aging, which highlights lipid metabolism as a potential therapeutic target for extending healthy lifespan in humans [1,2]. The liver is the central organ responsible for fatty acid and triglyceride (TG) metabolism through complex yet precisely coordinated regulatory pathways [3]. Dysregulation of these pathways contributes to diseases such as non-alcoholic fatty liver disease [3,4] and cancer [5]. Fatty acids accrue in the liver through cellular uptake from plasma and *de novo* synthesis [3,6]. Acetyl-CoA carboxylase 1 (ACC1) and fatty acid synthase (FASN) are essential enzymes that catalyze the rate-limiting steps of *de novo* fatty acid synthesis: ACC1 converts acetyl-CoA to malonyl-CoA, which is subsequently utilized by FASN [3,7,8]. In addition, ACC1 activity influences histone modification because acetyl-CoA serves as a key substrate for protein acetylation, including histones [[9], [10], [11], [12], [13], [14]]. These findings highlight the bidirectional interplay between lipid metabolism and epigenetic regulation. Furthermore, transcriptional activation by ACC1 and FASN requires sterol regulatory element-binding protein 1 (SREBP1), a master transcription factor that governs fatty acid synthesis [[15], [16], [17]].

Histones are the central components of the nucleosome, which comprises an octamer of histone proteins (two each of the core histone protein histones H2A, H2B, H3, and H4) wrapped around a 147-base-pair (bp) segment of DNA [18,19]. The core histone tails are often decorated with covalent post-translational modifications and cooperate to govern the chromatin state. This affects the epigenetic transcriptional regulation of various target genes [[18], [19], [20]]. Moreover, the biological roles of the linker histone H1 are less well characterized than those of the core histones. However, H1 has played essential roles in chromatin dynamics during the cell cycle [[21], [22], [23], [24]], transcriptional activation, and control of differentiation and senescence [[21], [22], [23], [24], [25], [26], [27]]. Despite extensive research, the regulation of individual histone proteins and their contribution to global physiological functions remain incompletely understood.

Inhibitor of DNA binding/differentiation (ID) 4-deficient mice previously lost body mass and died prematurely approximately three weeks after birth [28]. ID4 is a member of the ID family of proteins (ID1–4), which act as dominant negative regulators of basic helix-loop-helix (bHLH) transcription factors [[29], [30], [31], [32]]. However, ID4 exhibits a distinct tissue distribution and unique biological functions compared with those of ID1, ID2, and ID3 [[33], [34], [35], [36], [37], [38], [39], [40], [41], [42]]. Furthermore, ID4 is indispensable for the differentiation of several tissues, including adipose tissue [30,31,36], which is a major energy reservoir and an endocrine organ essential for systemic glucose and lipid homeostasis.

This study aimed to elucidate the role of ID4 in hepatic lipid homeostasis. Furthermore, beyond the classical view that ID4 is a negative regulator of bHLH transcription factors, the study investigated whether ID4 contributes to lipid metabolism through epigenetic mechanisms.

## 2 Materials and methods

### 2.1 Mice

*Id4*^*−/−*^ mice generated at the University of Nottingham (UK) [29] were provided by RIKEN BRC (Tsukuba, Japan) through the National BioResource Project of MEXT/AMED, Japan (RBRC04832). All animal experiments were approved by the Animal Ethics Committee of Kyushu University (Permission No. A22–055 and A24-043).

### 2.2 Histological analysis

Paraffin-embedded samples were subjected to hematoxylin and eosin (H&E) staining, and frozen tissue sections were stained with Oil Red O at the Biopathology Institute Co., Ltd. (Kunisaki, Japan).

### 2.3 Serum analysis

Determination of TG, cholesterol, and free fatty acid (FFA) levels in mouse serum was outsourced to Nagahama Life Science Laboratory of Oriental Yeast Co., Ltd. (Nagahama, Japan).

### 2.4 Hepatic lipid analysis

Lipids were extracted from liver tissues using the chloroform–methanol solvent extraction method. Frozen liver tissues were homogenized, placed in a 2:1 chloroform: methanol mixture, and mixed vigorously for 30 s. After centrifugation at 13,000 rpm for 15 min at 4 °C, the upper liquid phase was removed. Hepatic TG and FFA concentrations were measured using LabAssay Triglycerides (Fujifilm Wako, Osaka, Japan) and LabAssay NEFA (Fujifilm Wako) kits, respectively.

### 2.5 Comprehensive transcriptomic analysis

RNA sequencing (RNA-seq) was performed using MGI DNBSEQ-G400 FAST (MGI Tech, Shenzhen, China). RNA samples were quantified using an ND-1000 spectrophotometer (NanoDrop), and their quality was confirmed using an Agilent 2200 TapeStation system (Agilent, Santa Clara, CA, USA). After rRNA depletion using the MGIEasy rRNA Depletion kit (MGI Tech), RNA directional libraries for MGI DNBSEQ-G400 FAST were prepared using the MGIEasy RNA Directional Library Prep Set (MGI Tech) according to the manufacturer's instructions. To identify up- or down-regulated genes, Z-scores and non-log-scaled fold-change ratios were calculated from the normalized signal intensities of each probe. The criteria for up- or down-regulated genes were as follows: up-regulated genes, Z-score ≥2.0 and ratio ≥1.5-fold; down-regulated genes, Z-score ≤ −2.0 and ratio ≤0.66.

### 2.6 Comprehensive chromatin accessibility analysis

Assay for transposase-accessible chromatin sequencing (ATAC-seq) was performed on three biological replicates for *Id4*^*+/+*^ and *Id4*^*−/−*^ conditions. Raw paired-end reads were subjected to adapter trimming and quality filtering using fastp v.0.23.4 [43], while allowing up to 10% of bases with <Q20, correction of mismatched bases in overlapping regions, poly A tail trimming, and automatic detection of adapter sequences in the paired-end mode. Reads shorter than 25 bp or those failing quality requirements were discarded. The trimmed reads were mapped to the *Mus musculus* reference genome (mm10) using bowtie2 v.2.5.3 [44]. Alignment was performed in end-to-end mode with the very-sensitive preset, while disallowing mixed or discordant pairings and enabling dovetail alignments. Aligned reads were processed using Samtools v.1.20 [45]. Reads with a mapping quality of <Q20 or improper pairing were excluded. The BAM files were then sorted and indexed. Polymerase chain reaction (PCR) duplicates were removed using Picard MarkDuplicates, and reads overlapping with the ENCODE mm10 blacklist regions were excluded. Only uniquely mapped, high-quality, and non-duplicate reads were retained for downstream analysis. Accessible chromatin regions (peaks) were identified using MACS2 v.2.2.7.1 [46] with a fixed fragment length of 150 bp. To assess differential chromatin accessibility between wild-type and *Id4*-knockout samples, the specific Peaks py function from the ATACgraph package was used [47] based on three biological replicates. Differentially accessible regions were annotated using ChIPseeker [48] by focusing on peaks located within the promoter and enhancer regions. *Mus musculus* liver-specific enhancer annotations were retrieved from the EnhancerAtlas 2.0 database [49]. Visualization of chromatin accessibility patterns for differentially accessible regions were generated using deepTools v.3.5.1 modules bamCoverage, computeMatrix, and plotHeatmap [50].

### 2.7 Comprehensive histone modification analysis

Liver tissues were outsourced to Active Motif (Carlsbad, CA, USA; ModSpec® platform), where histones were extracted, propionylated, and digested with trypsin prior to mass spectrometry analysis using a triple quadrupole instrument. The relative abundance of each histone modification was determined using the provider's standardized analysis pipeline.

### 2.8 Liquid-chromatography tandem mass-spectrometry analysis

To identify the molecules obtained by immunoprecipitation, targeted proteins separated via sodium dodecyl sulfate–polyacrylamide gel electrophoresis (SDS-PAGE) after silver staining were excised and outsourced to Japan Proteomics (Sendai, Japan). The proteins were identified using the provider's standardized analysis pipeline with a proteomic nano liquid chromatography tandem mass spectrometry (LC-MS/MS) system. For measurement of carnitine, acetylcarnitine, and β-hydroxybutyrate, LC-MS/MS analysis was performed using an LCMS-8050 system (Shimadzu, Kyoto, Japan). Carnitine, acetylcarnitine, and β-hydroxybutyrate, were analyzed using the Shimadzu LC/MS/MS Method Package for Primary Metabolites or Short Chain Fatty Acids, respectively. Liver tissues were extracted using 0.1% formic acid-containing solvent with the corresponding internal standard, followed by acetonitrile extraction and 3-kDa ultrafiltration. For β-hydroxybutyrate analysis, samples were further derivatized with 3-nitrophenylhydrazine before LC-MS/MS analysis. Carnitine and acetylcarnitine or β-hydroxybutyrate signals were normalized to 2-(*N*-morpholino) ethanesulfonic acid or 2-ethylbutyric acid, respectively. The normalized values were further adjusted to tissue weight.

### 2.9 Reverse transcription-quantitative polymerase chain reaction analysis

Quantitative PCR analysis was performed using the same methods as previously described [51]. The cycling conditions were as follows: 95 °C for 10 min, followed by 40 cycles of 95 °C for 5 s and 60 °C for 30 s, using the PowerUp SYBR Green Master Mix (Thermo Fisher Scientific). PCR primer sequences are listed in Supplemental Table 1.

### 2.10 Immunoprecipitation

Liver tissues were dissected from three-week-old male mice and homogenized in RIPA buffer (Nacalai Tesque) to prepare tissue lysates. For immunoprecipitation, 150 μg of tissue lysates were incubated at 4 °C for 2 h with Dynabeads Protein G (Thermo Fisher Scientific) bound to anti-ID4 antibody (Santa Cruz Biotechnology, Dallas, TX). The precipitated proteins were subjected to SDS-PAGE for silver staining using a Silver Stain MS kit (Fujifilm Wako) or immunoblot analysis with anti-histone H1 antibody (Santa Cruz Biotechnology).

### 2.11 Western blot analysis

Western blot analysis was performed using the same methods as previously described [51], with primary antibodies (Supplemental Table 2) followed by incubation of secondary antibodies conjugated to horseradish peroxidase (Cell Signaling Technology). For detection of immunoprecipitated proteins, the VeriBlot for IP Detection Reagent (diluted 1:1,000, Cat#ab131366; Abcam), a secondary antibody specific to the native (non-reduced) antibody, was used. Blots were analyzed using an ImageQuant LAS4000 system (GE Healthcare, Chicago, IL, USA) with Chemi-Lumi One (Nacalai Tesque).

### 2.12 Chromatin accessibility assay

Chromatin was purified from liver tissues using an EpiQuik Chromatin Accessibility Assay kit (Epigentek, Farmingdale, NY, USA), according to the manufacturer's instructions. Quantitative PCR was performed using PowerUp SYBR Green Master Mix and the StepOnePlus real-time PCR system (Thermo Fisher Scientific). The cycling conditions were as follows: 95 °C for 10 min, followed by 40 cycles of 95 °C for 5 s and 59 °C for 30 s. The LXR: RXR-binding site in the *Srebf1* promoter was identified using the JASPAR database, and PCR primers were designed to flank this region. The PCR primer sequences were 5′-AACAGAGGTCCTGAGGACCT-3′ and 5′-CACAGGGCCCAGGGTTCACTT-3’.

### 2.13 Isolation of liver mitochondria and oxygen consumption assay

Liver mitochondria were isolated using an IntactMito Fractionation Kit for Tissue (MT17; Dojindo Laboratories, Kumamoto, Japan) according to the manufacturer's instructions. Oxygen consumption rate (OCR) was assessed using an Extracellular OCR Plate Assay Kit (E297; Dojindo Laboratories) according to the manufacturer's instructions. Calculated OCR values were determined from time-dependent changes in oxygen-sensitive fluorescence using the manufacturer's calculation template.

### 2.14 Statistical analysis

Student's *t*-tests were performed as appropriate. All quantitative data are presented as means ± standard error of the mean. *P* values < 0.05 were considered statistically significant.

## 3 Results

### 3.1 Aberrant adipocyte differentiation and serum lipid profiles in Id4^−/−^ mice

**Figure 1:**
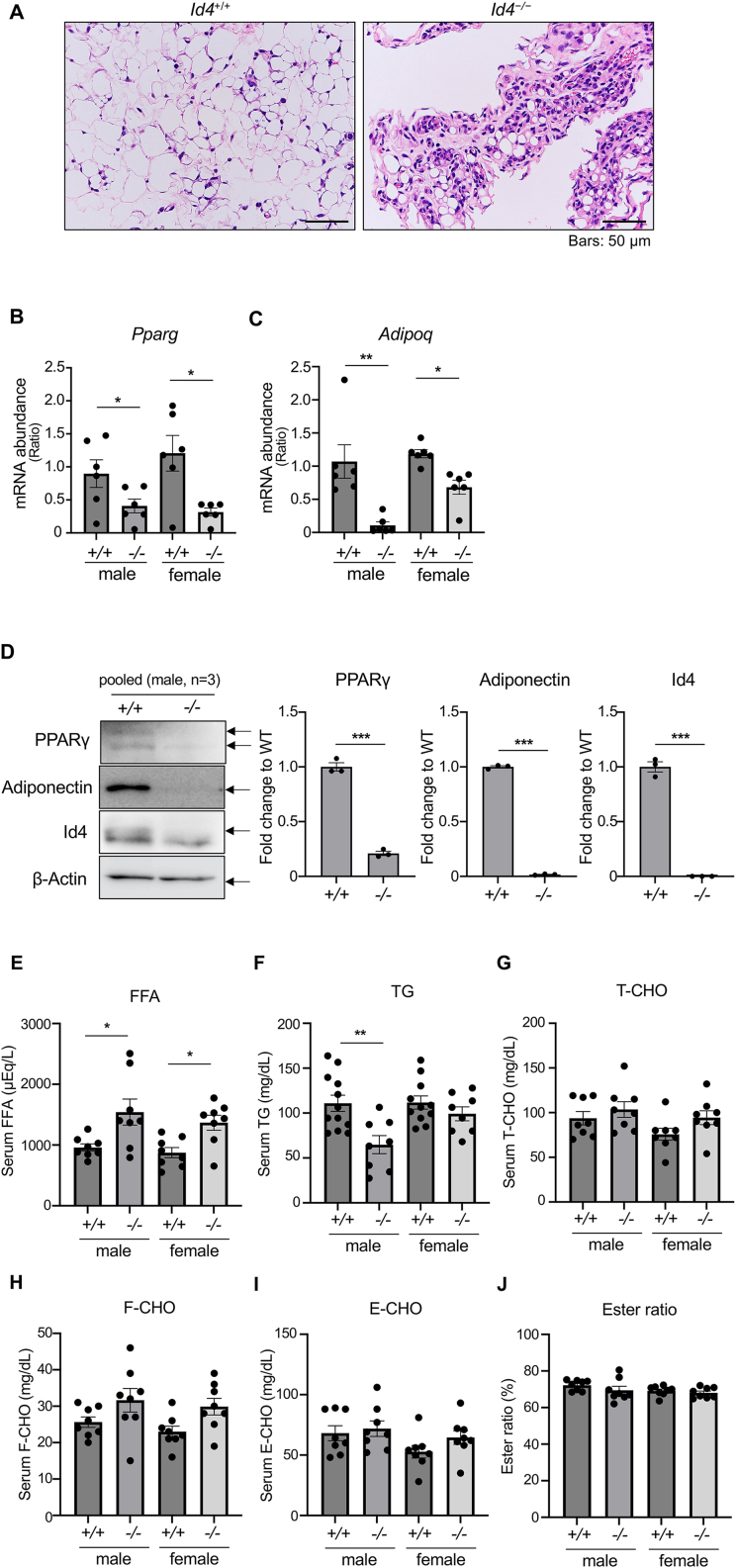
**Characterization of white adipose tissues and serum lipid profiles in Id4^−/−^ mice at three weeks of age**. (A) Representative images of hematoxylin and eosin (HE) staining of gonadal white adipose tissues in *Id4*^*+/+*^ (left) and *Id4*^*−/−*^ (right) three-week-old male mice. Reverse transcription-quantitative polymerase chain reaction (RT-qPCR) for (B) *Pparg* and (C) *Adipoq* in gonadal white adipose tissues. (D) Western blot analysis of gonadal white adipose tissues performed using three independent pooled samples per group, with each pooled sample consisting of adipose tissues from three mice (total n = 9 mice per group). Representative image (left) and densitometric quantification of protein levels normalized to the corresponding loading controls (right). (E) Free fatty acid (FFA), (F) triglycerides (TG), (G) total cholesterol (T-CHO), (H) free cholesterol (F–CHO), and (I) esterified cholesterol (E-CHO) were measured in the serum of *Id4*^*+/+*^ and *Id4*^*−/−*^ three-week-old mice. (J) Fractional cholesterol esterification was calculated as the ratio of E-CHO to T-CHO in each serum sample. Data are presented as mean ± standard error of the mean (SEM). Student's t-test (two-tailed) was used for statistical comparisons, performed separately for males and females. ∗*P* < 0.05, ∗∗*P* < 0.01, and ∗∗∗*P* < 0.001.

Based on previous results showing that ID4 is involved in adipocyte differentiation [30,32,36], this study first examined the adipose tissue of *Id4*^*−/−*^ mice and found that the number of adipocytes filled with TGs was markedly reduced in *Id4*^*−/−*^ mice compared with that in *Id4*^*+/+*^ mice (Figure 1A). This observation was further supported by the significantly reduced expression of adipocyte differentiation markers, such as peroxisome proliferator-activated receptor γ and adiponectin in *Id4*^*−/−*^ mice (Figure 1B–D). Given that adipose tissue critically maintains systemic fat metabolism [52], serum lipid species were measured in these mice. The results showed that serum FFA concentrations were markedly elevated in *Id4*^*−/−*^ mice compared with that in *Id4*^*+/+*^ littermates (Fig. 1E). Serum TG levels were significantly decreased in *Id4*^*−/−*^ male mice compared with that in wild-type controls, whereas no significant difference was observed in females (Fig. 1F). Moreover, no notable differences in serum cholesterol levels or cholesterol esterification rates were observed between *Id4*^*+/+*^ and *Id4*^*−/−*^ mice (Figure 1G–J). Considering that fatty acid metabolism is most active in hepatocytes [1,3], the study next focused on the liver to elucidate the role of ID4 in lipid metabolism.

### 3.2 Fatty acid metabolism dysregulation in Id4^−/−^ mouse liver

**Figure 2:**
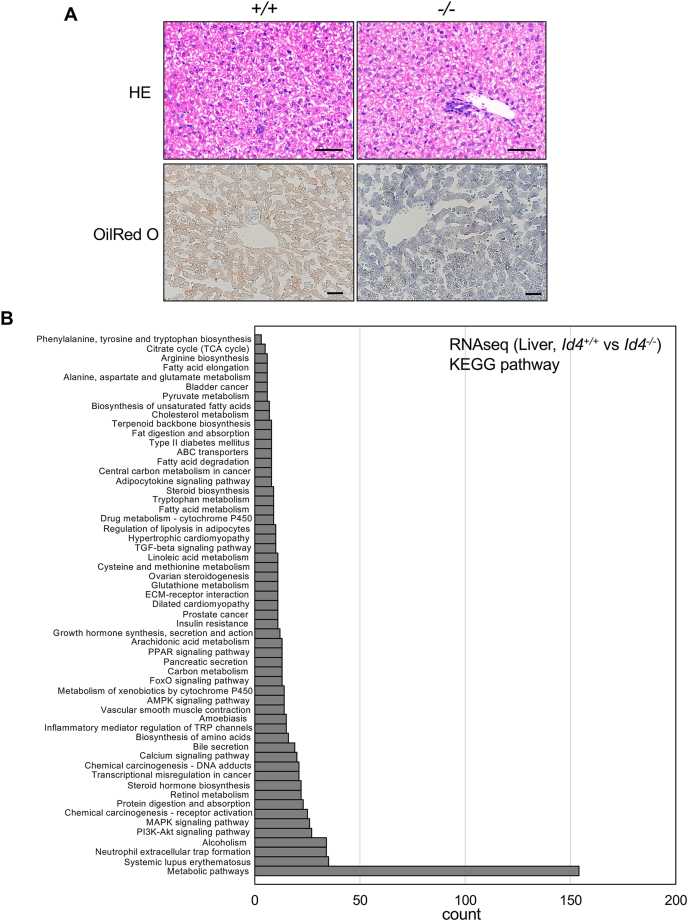

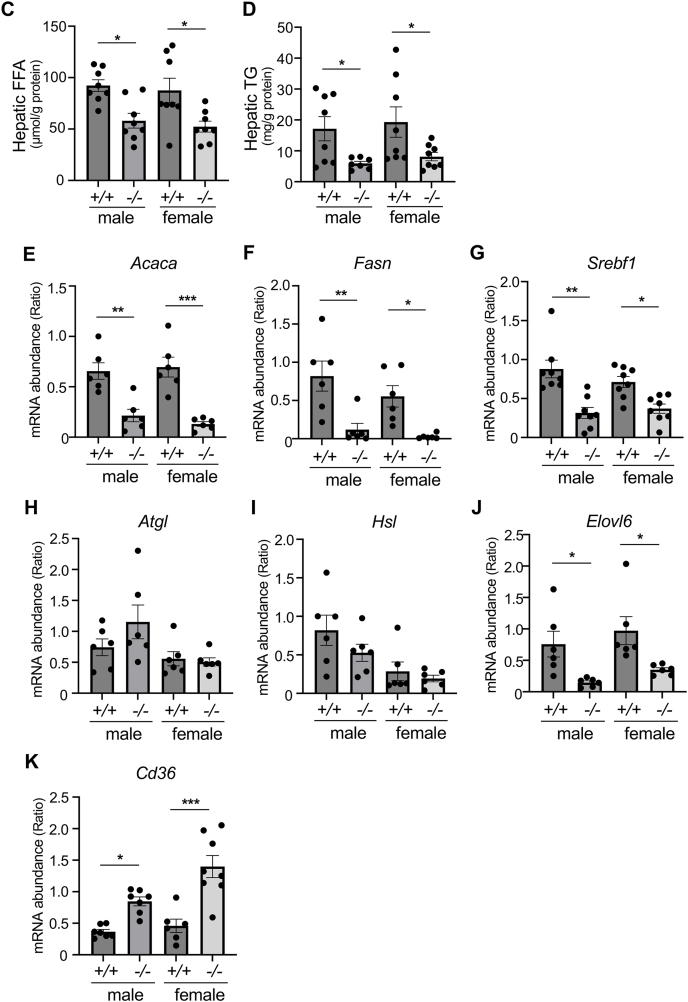

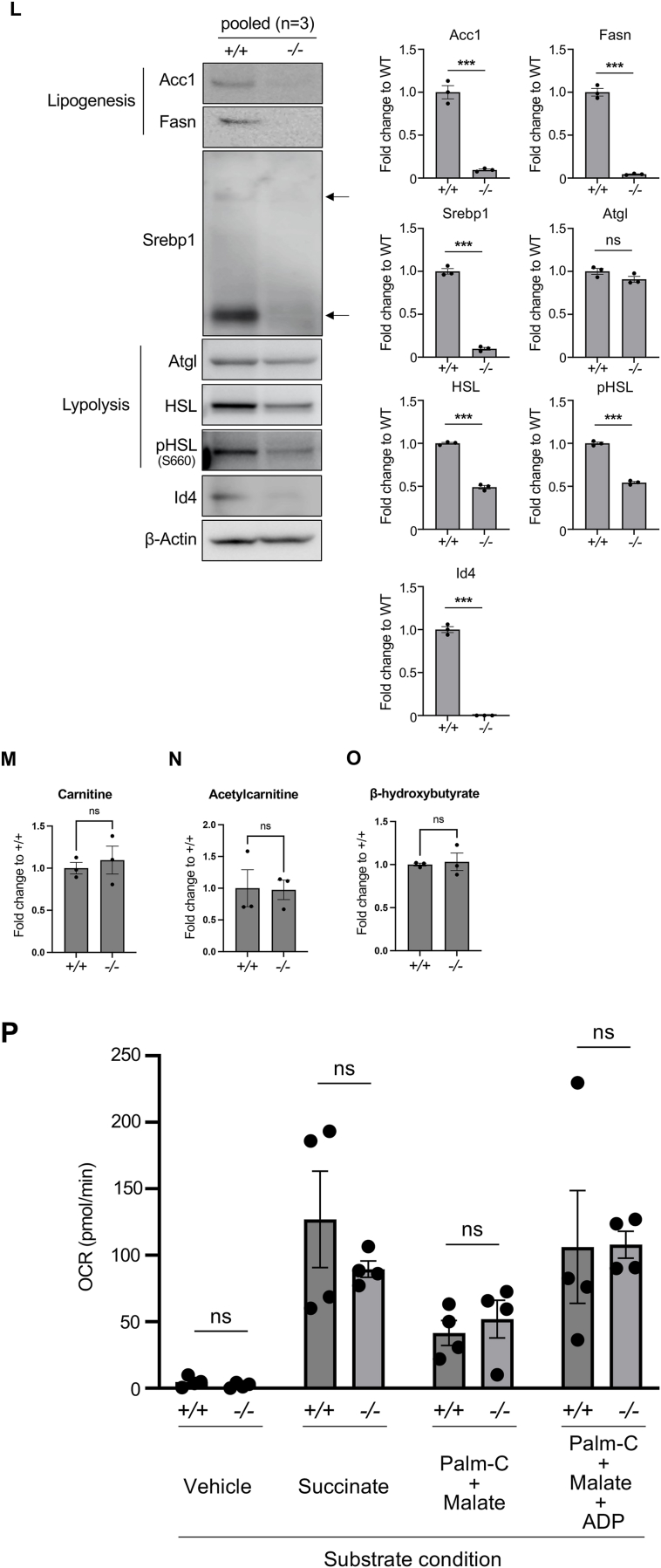
**Characterization of liver from Id4^−/−^ mice at three weeks of age**. (A) Representative images of hematoxylin and eosin (HE, upper images) and Oil Red O staining (lower images) of the liver tissues in *Id4*^*+/+*^ (left) and *Id4*^*−/−*^ (right) mice. (B) Kyoto Encyclopedia of Genes and Genomes (KEGG) enrichment analyses of genes differentially expressed in the liver tissues of *Id4*^*+/+*^ and *Id4*^*−/−*^ mice (n = 3) from RNA sequencing (RNAseq) analysis. The vertical coordinate shows the name of the KEGG metabolic pathway, whereas the horizontal coordinate shows the number of transcripts annotated to the pathway. Quantitative analyses of (C) hepatic free fatty acids (FFA) and (D) triglycerides TG. Reverse transcription-quantitative polymerase chain reaction (RT-qPCR) analysis for (E) *Acaca*, (F) *Fasn*, (G) *Srebf1*, (H) *Atgl*, (I) *Hsl*, (J) *Elovl6*, and (K) *Cd36* in the liver. (L) Western blot analyses of acetyl-CoA carboxylase 1 (ACC1), fatty acid synthase (FSN), sterol regulatory element-binding protein 1 (SREBP1), adipose triglyceride lipase (ATGL), hormone-sensitive lipase (HSL), phospho-HSL (S660), and ID4 in *Id4*^*+/+*^ and *Id4*^*−/−*^ liver tissues performed using three independent pooled samples per group, with each pooled sample consisting of liver tissues from three mice (total n = 9 mice per group). β-actin was used as an internal control. Representative images are shown (left), and densitometric quantification of protein levels normalized to β-Actin are shown (right). Mass spectrometry analysis for (M) carnitine, (N) acetylcarnitine, and (O) β-hydroxybutyrate in liver tissues of *Id4*^*+/+*^ and *Id4*^*−/−*^ mice (n = 3). (P) Calculated OCR of isolated liver mitochondria from *Id4*^*+/+*^ and *Id4*^*−/−*^ mice (n = 4). Isolated mitochondria were plated at 50 μg mitochondria per well and incubated under the indicated substrate conditions: vehicle, succinate, palmitoyl-L-carnitine plus malate, or palmitoyl-L-carnitine plus malate and ADP. The final concentrations were as follows: succinate, 10 mM; palmitoyl-L-carnitine, 10 μM; malate, 0.2 mM; and ADP, 150 μM. Palm-C/Malate indicates palmitoyl-L-carnitine plus malate. Data are presented as mean ± standard error of the mean (SEM). Student's t-test (two-tailed) was used for statistical comparisons, performed separately for males and females. ∗*P* < 0.05, ∗∗*P* < 0.01, and ∗∗∗*P* < 0.001, ns: not significant.

Liver tissues were first examined histologically using H&E staining, which revealed no morphological differences between *Id4*^*+/+*^ and *Id4*^*−/−*^ livers (Figure 2A). Next, comprehensive transcriptomic analysis was performed to identify ID4-dependent alterations in hepatic gene expression (Supplemental Table 3, Fig. 2B). Kyoto Encyclopedia of Genes and Genomes (KEGG) enrichment analysis showed that differentially expressed genes were significantly enriched in metabolic pathways related to fatty acid homeostasis, including fatty acid elongation, biosynthesis of unsaturated fatty acids, fatty acid degradation, and fatty acid metabolism (Fig. 2B). Therefore, hepatic FFA and TG levels in *Id4*^*+/+*^ and *Id4*^*−/−*^ mice were measured. Hepatic FFA and TG levels were markedly reduced in *Id4*^*−/−*^ mice compared with those in their *Id4*^*+/+*^ littermates (Fig. 2C–D). These findings were further supported by Oil Red O staining results, which demonstrated reduced lipid accumulation in *Id4*^*−/−*^ liver tissues (Fig. 2A). Because the liver is involved in the uptake, synthesis, storage, secretion, and catabolism of fatty acids and TGs [1,3], the expression of genes involved in hepatic fatty acid metabolism was examined. Key fatty acid synthesis-related molecules, including *Acaca* (encoding ACC1), *Fasn*, and *Srebf1* (encoding SREBP1) were significantly downregulated in *Id4*^*−/−*^ livers (Fig. 2E–G). In contrast, no consistent patterns were observed in terms of the expression or activation of fatty acid degradation-related genes, such as those encoding adipose TG lipase and hormone-sensitive lipase (HSL) (Fig. 2H–I). Fatty acid elongase 6, a microsomal enzyme that elongates saturated and monosaturated fatty acids [53], was also significantly reduced in *Id4*^*−/−*^ livers (Fig. 2J). CD36, which promotes fatty acid uptake by hepatocytes [1,3], was upregulated in *Id4*^*−/−*^ mice (Fig. 2K). At the protein level, the results were generally consistent with their mRNA expression patterns; however, HSL protein levels were decreased in *Id4*^*−/−*^ livers (Fig. 2L). Furthermore, additional mass spectrometry analyses showed that carnitine, acetylcarnitine, and β-hydroxybutyrate levels were comparable between *Id4*^*+/+*^ and *Id4*^*−/−*^ livers (Fig. 2M–O). We also performed OCR measurements using mitochondria isolated from *Id4*^+/+^ and *Id4*^−/−^ liver tissues. No significant differences in calculated OCR were observed under the indicated substrate conditions, including succinate and palmitoyl-L-carnitine/malate with or without ADP (Fig. 2P). These findings suggest that no apparent changes in substrate-supported mitochondrial respiration were detected in *Id4*^−/−^ liver mitochondria under the conditions examined. Together with the comparable levels of carnitine, acetylcarnitine, and β-hydroxybutyrate, these data did not provide evidence for marked alterations in fatty acid catabolism under the conditions examined.

### 3.3 ID4 is required to maintain normal chromatin dynamics in liver

**Figure 3:**
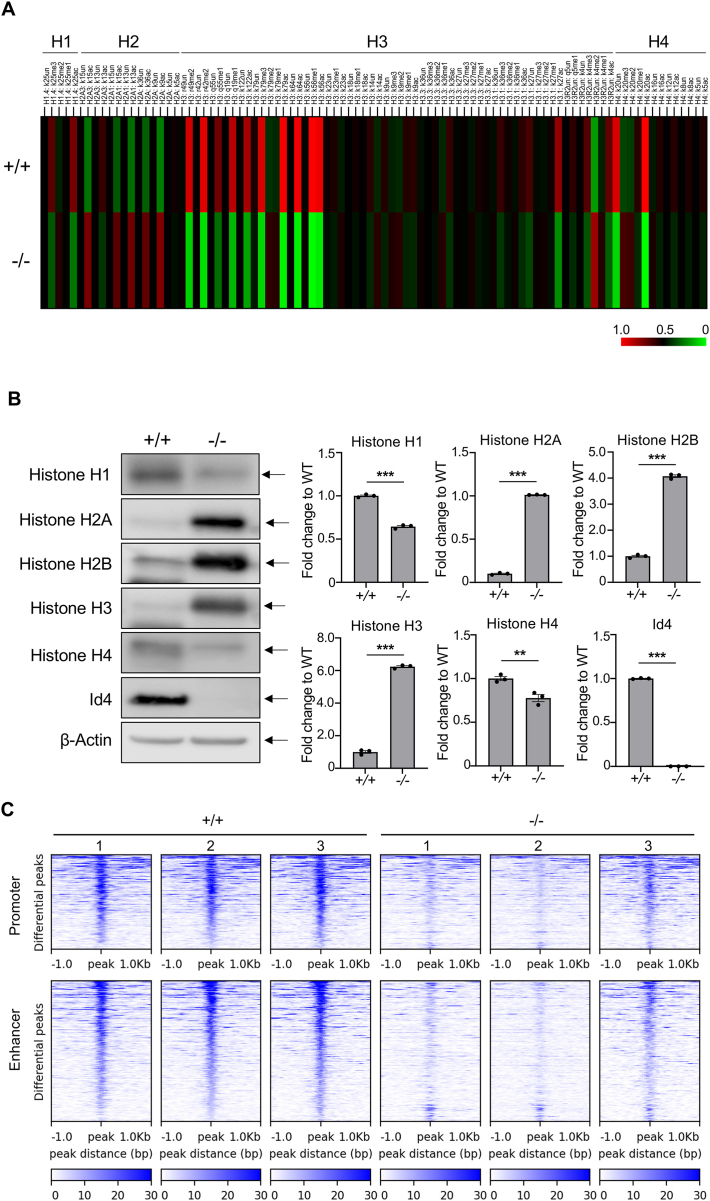

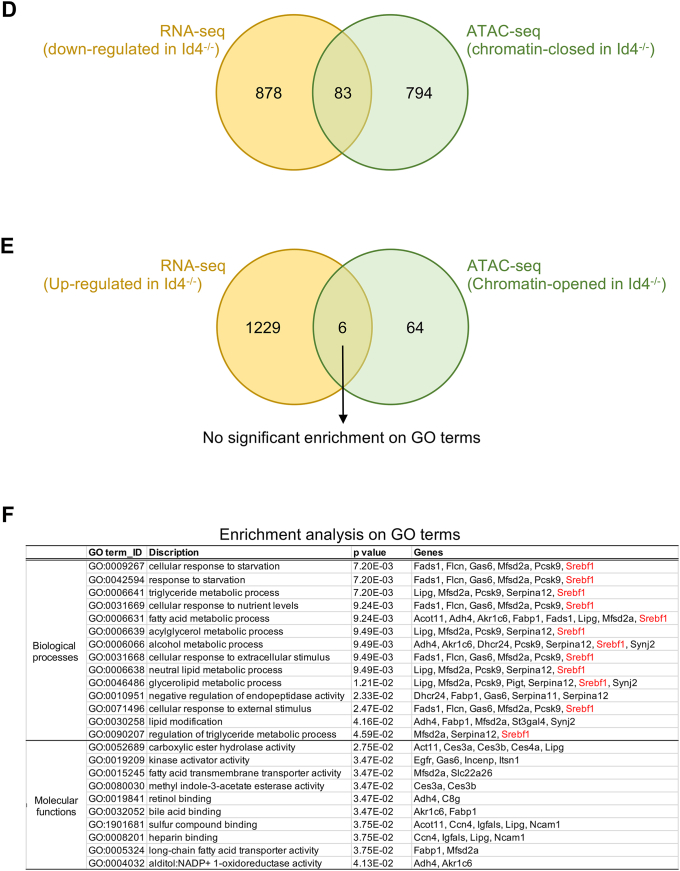

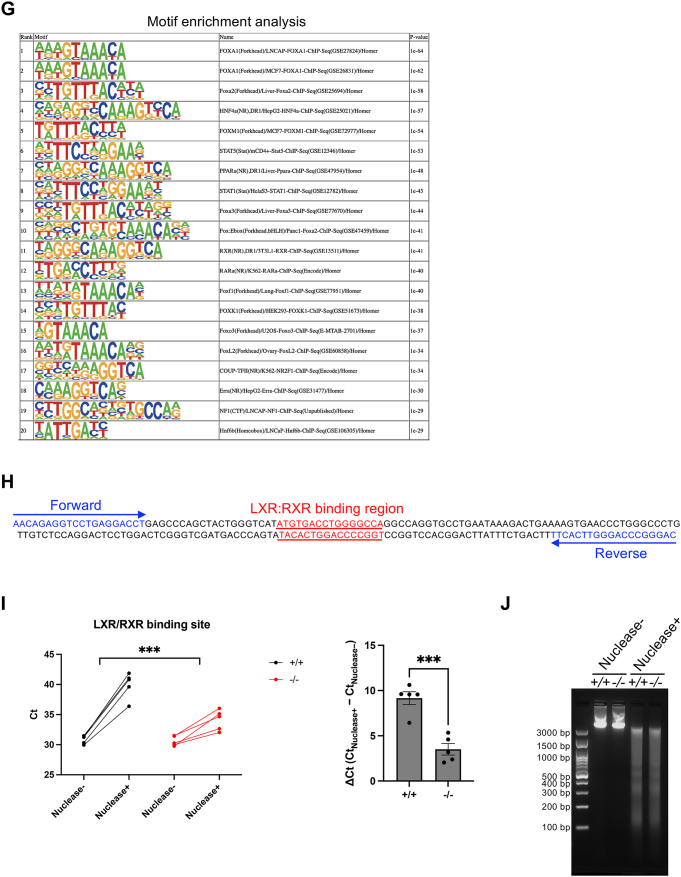
**Aberrant histone expression and modification patterns in Id4^−/−^ male mouse liver**. (A) A heatmap of Modspec® analysis in *Id4*^*+/+*^ and *Id4*^*−/−*^ three-week-old male mice. (B) Western blot analysis for histones H1 (33 kDa), H2A (14 kDa), H2B (14 kDa), H3 (17 kDa), H4 (11 kDa), and ID4 (18 kDa) in *Id4*^*+/+*^ and *Id4*^*−/−*^ male mouse liver tissues performed using three independent pooled samples per group, with each pooled sample consisting of liver tissues from three mice (total n = 9 mice per group). β-actin was used as an internal control. Representative images are shown (left), and densitometric quantification of protein levels normalized to β-actin is shown as bar graphs (right). (C–G) Changes in chromatin accessibility landscape of mouse hepatocytes. (C) Assay for transposase-accessible chromatin (ATAC) signals. For integrative analysis, ATAC-seq data were combined with RNA sequencing (RNA-seq) profiles. After two categories of genes were selected, (D) genes that were both substantially downregulated in RNA-seq and associated with reduced chromatin accessibility in ATAC-seq in *Id4*^*−/−*^ samples were identified. (E) Similarly, genes upregulated in RNA-seq and showing increased chromatin accessibility were extracted. (F) Functional enrichment of these gene sets was assessed via Gene Ontology (GO) overrepresentation analysis (ORA) using the R package clusterProfiler [62] to infer biological processes potentially regulated by ID4. (G) Motif enrichment within differential accessible regions was analyzed using HOMER [63] to investigate transcriptional regulation. Based on the integrated results, candidate regulator SREBF1 was selected for further experimental validation. (H–I) Chromatin accessibility assay. Primer sequences flanking the LXR:RXR binding region in the promoter of *Srebf1* gene (H), and Ct values in the presence and absence of nuclease treatment (left) and ΔCt values (nuclease-treated minus untreated) (right) obtained from qPCR analysis (I). (J) Representative agarose gel image showing the extent and efficiency of MNase digestion across samples, confirming comparable digestion conditions. Data are presented as mean ± standard error of the mean (SEM). Student's t-test (two-tailed) was used. ∗*P* < 0.05, ∗∗*P* < 0.01, and ∗∗∗*P* < 0.001.

**Figure 4:**
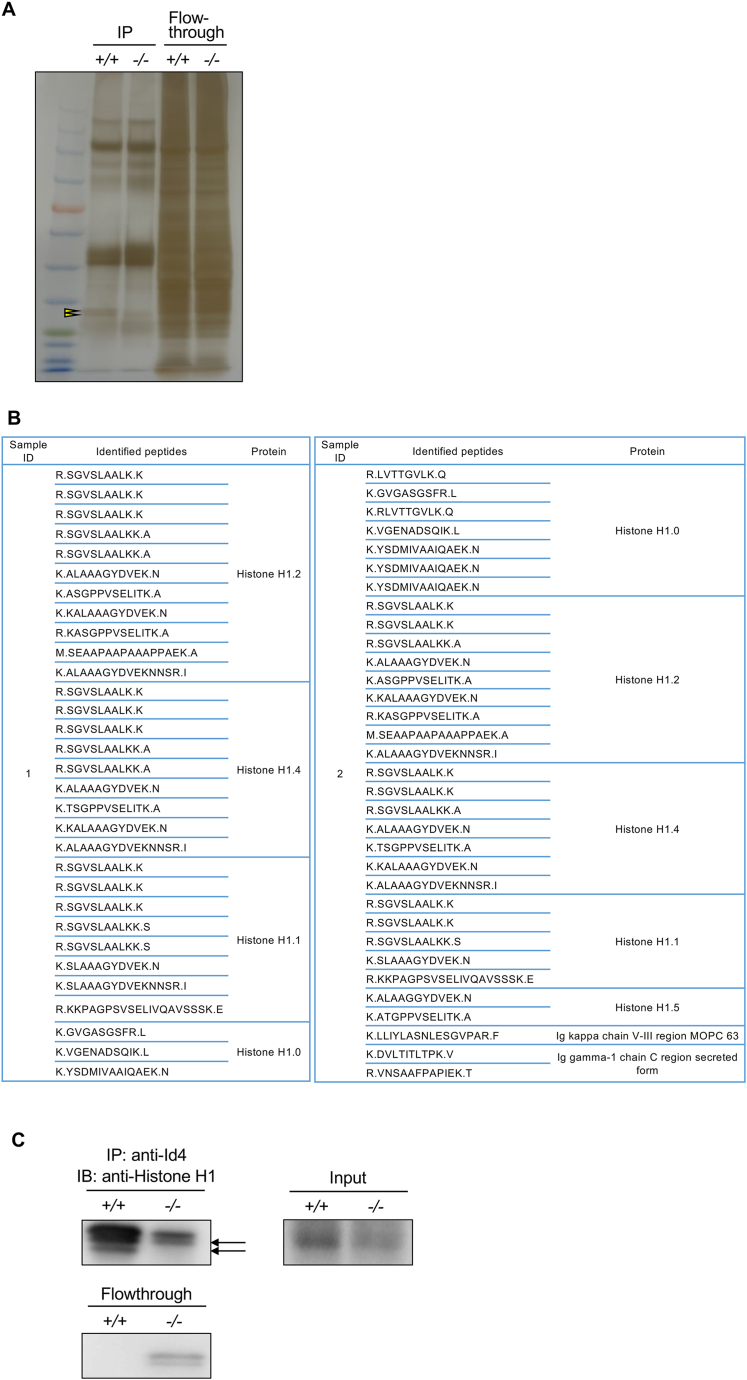
**Direct binding between ID4 and Histone H1 in an immunoprecipitation assay**. (A) A representative silver-stained sodium dodecyl sulfate–polyacrylamide gel electrophoresis (SDS–PAGE) image of liver tissue proteins immunoprecipitated with an anti-ID4 antibody from three-week-old male *Id4*^*+/+*^ and *Id4*^*−/−*^ male mice. Arrows indicate Histone H1 proteins validated by mass spectrometry. (B) Peptide sequences obtained by mass spectrometry from the bands indicated by arrowheads in (A) (upper: sample No.1; lower: sample No.2), along with the corresponding proteins identified based on sequence matching. (C) Immunoblot analysis of the proteins immunoprecipitated with an anti-ID4 antibody for Histone H1.

ACC1 regulates global histone acetylation, thereby providing a mechanistic link between fatty acid metabolism and epigenetic regulation of transcription [11,12]. Therefore, a comprehensive analysis of histone modifications in male mouse liver tissues was performed. Because the phenotypes were largely similar between male and female mice (Figure 1, Figure 2), subsequent analyses were conducted using male mice to further elucidate the mechanisms underlying the reduced hepatic FFA levels. Mass spectrometry profiling revealed broad alterations involving multiple histone modifications in *Id4*^*−/−*^ livers, including changes in several active markers, such as H3K27ac, H3K4me3, H3K56ac, and H3K79me3. This indicated that the normal modification landscape associated with transcriptionally active chromatin was disrupted (Fig. 3A). Western blot analysis showed the selective accumulation of core histones (H2A, H2B, and H3), and these changes in histone protein levels may influence the interpretation of the observed alterations in histone modifications (Fig. 3B). ATAC-seq profiling further demonstrated a marked change in chromatin accessibility at both promoters and enhancers in *Id4*^*−/−*^ liver nuclei, with diminished and less sharply defined accessibility peaks across all biological replicates (Fig. 3C). In addition, no pronounced differences were observed in the expression levels of histone deacetylases (HDAC) (Fig. S1A), and histone acetyltransferases (HAT) (Fig. S1B), which regulate histone acetylation [54] between *Id4*^*+/+*^ and *Id4*^*−/−*^ livers. Furthermore, the levels of hepatic acetyl-CoA, which is a key substrate for histone acetylation [11], were comparable between the two genotypes (Fig. S1C). Considering that IDs typically function by interacting with bHLH proteins and modulating E-box-binding transcription factors, immunoprecipitation was performed to explore potential protein interactions using an anti-ID4 antibody followed by silver staining. [32,33]. Surprisingly, silver staining (Figure 4A) and subsequent mass spectrometry analysis (Fig. 4B) of ID4-associated protein bands present only in *Id4*^*+/+*^ samples indicated that ID4 interacts with histone H1, a non-bHLH protein. This interaction was further validated via immunoblot analysis using anti-histone H1 antibody that recognizes multiple H1 variants [22,27] (Fig. 4C). Although the structural and functional roles of linker histone H1 in chromatin architecture remain unclear, histone H1 has promoted higher-order chromatin organization and compaction [22,55]. Next, ATAC-seq data was integrated with RNA-seq results (Fig. 3DE, 2B). Integrative analysis identified *Srebf1* among the genes that were downregulated in RNA-seq and which exhibited chromatin closure by ATAC-seq (Fig. 3DF). Motif enrichment analysis further revealed enrichment of the RXR-binding motif, which is essential for *Srebf1* transcriptional activation [[56], [57], [58]] (Fig. 3G). In contrast, the genes that were upregulated in RNA-seq and displayed open chromatin states in ATAC-seq showed no significant Gene Ontology term enrichment (Fig. 3E). To independently assess chromatin accessibility, DNA was purified from nuclease-treated and untreated chromatin preparations, and qPCR targeting the LXR:RXR-binding region was performed within the *Srebf1* promoter. The micrococcal nuclease (MNase) digestion efficiency and nuclease-dependent Ct shift was markedly smaller in *Id4*^*−/−*^ samples than in *Id4*^*+/+*^ controls (Fig. 3H–J), thereby indicating reduced chromatin accessibility. These findings suggest that in the livers of *Id4*^*−/−*^ mice, the LXR:RXR binding region essential for *Srebf1* activation adopts a heterochromatic and transcriptionally restrictive state. Collectively, these results indicated that impaired hepatic fatty acid synthesis in *Id4*^*−/−*^ mice is driven by reduced chromatin accessibility at the *Srebf1* promoter as a consequence of ID4 deficiency.

## 4 Discussion

This study was initiated to elucidate the mechanisms underlying premature death observed in *Id4*^*−/−*^ mice [28,29], which is likely attributable to abnormalities in energy metabolism. ID4 plays important roles in the differentiation of multiple tissues including adipose tissue [30,32,36], submandibular glands [28], mammary glands [33,37], osteoblasts [31], and the central nervous system [29]. Numerous adipocytes lacking TG stores in *Id4*^*−/−*^ adipose tissue reflects impaired adipocyte differentiation in such mice [30,32,36]. This study explored the possibility that ID4 contributes to systemic energy metabolism, particularly hepatic lipid metabolism. Given the significant differences in hepatic FFA levels between *Id4*^*+/+*^ and *Id4*^*−/−*^ mice, the study hypothesized that ID4 may regulate the transcription of upstream factors governing fatty acid synthesis, uptake, and metabolism. However, our analysis revealed that ID4 deficiency is associated with alterations in histone dynamics, suggesting that epigenetic dysregulation accompanies the observed defects in lipid metabolism. In the *Id4*^*−/−*^ liver, both histone modifications and expression levels of multiple histone proteins were dysregulated. Furthermore, the *in vitro* analyses demonstrated reduced histone H1 expression in *Id4*^*−/−*^ livers and revealed a physical interaction between ID4 and linker histone H1. Although the function of histone H1 is not fully understood, its expression is tightly regulated during the S phase in a replication-dependent manner [54,59] and is involved in senescence [25], differentiation [24], cancer progression [23], and transcriptional regulation. A marked reduction in histone H1 levels also markedly alters organismal lifespan [[25], [26], [27]]. Given the involvement of ID4 in differentiation [[28], [29], [30], [31], [32], [33], [34], [35], [36], [37]], cellular senescence or aging [35,60], and cancer [33,35,38,39], the interaction between ID4 and histone H1 may contribute to these biological processes. Moreover, dysregulated core histone expression may further impact chromatin architecture. ATAC-seq analysis revealed marked alterations in both heterochromatin and euchromatin landscapes in *Id4*^*−/−*^ livers compared with those in *Id4*^*+/+*^ controls. Notably among these global changes, reduced chromatin accessibility was observed at the *Srebf1* promoter. SREBP1 is a central regulator of hepatic lipogenesis, and its downregulation was accompanied by decreased expression of the downstream target genes, including *Fasn* and *Acaca*. Therefore, these findings are consistent with the possibility that altered chromatin accessibility at *Srebf1* contributes to reduced lipogenic gene expression. However, the chromatin alterations observed had a genome-wide nature, and KEGG enrichment analysis of the RNA-seq data revealed changes in metabolic pathways beyond the fatty acid synthesis pathway. Therefore, the changes at the *Srebf1* locus could be interpreted as part of a broader epigenetic and transcriptional reprogramming rather than a single promoter-specific mechanism. In our chromatin accessibility assay (Fig. 3I–J), the positive control, the *β-actin* promoter region, which is generally considered to be constitutively active, showed comparable ΔCt values between *Id4*^*+*^*/*^*+*^ and *Id4*^*−/−*^ livers. In contrast, the negative control region, Untr6, which is generally considered to be constitutively inactive, exhibited markedly different ΔCt values between *Id4*^*+*^*/*^*+*^ and *Id4*^*−/−*^ livers. Therefore, it will be important to investigate how ID4 deficiency affects promoter-specific chromatin states across a broad range of loci in future studies. Furthermore, in this study, the expression of major HDACs, sirtuins, and HATs, as well as hepatic acetyl-CoA levels did not differ between *Id4*^*+*^*/*^*+*^ and *Id4*^*−/−*^ mice. Although these findings do not support a major contribution of altered enzyme expression or bulk hepatic acetyl-CoA availability to the observed changes in histone acetylation, some limitations should be considered. The acetyl-CoA measurements represent bulk tissue levels and do not assess subcellular acetyl-CoA pools, particularly within the nucleus, where histone acetylation is primarily regulated. Therefore, the possibility that localized metabolic changes contribute to the observed alterations in histone acetylation cannot be completely excluded. Nevertheless, our results suggest that the mechanisms underlying altered histone acetylation in *Id4*^*−/−*^ livers may involve regulatory processes beyond simple changes in enzyme expression or overall substrate availability.

The study has some limitations. First, the precise timing and structural context in which ID4 functions within the chromatin architecture *in situ* were not determined. Considering that intracellular chromatin exists as a highly heterogeneous and dynamic complex composed of numerous molecular components, isolating intact chromatin and analyzing its structural and biophysical properties *in vitro* remains technically challenging [61]. Second, mitochondrial fatty acid oxidation was not comprehensively evaluated in the present study. Although metabolite analyses, including carnitine, acetylcarnitine, and β-hydroxybutyrate measurements, as well as direct oxygen consumption rate analyses using isolated liver mitochondria, did not reveal substantial differences between *Id4*^*+*^*/*^*+*^ and *Id4*^*−/−*^ mice, these assessments do not fully capture fatty acid oxidation flux. Therefore, while our findings do not support the presence of marked mitochondrial dysfunction in *Id4*^*−/−*^ liver tissue, the possibility that alterations in fatty acid oxidation contribute to the observed phenotype cannot be completely excluded and should be examined in further studies, such as comprehensive acylcarnitine profiling. Third, another limitation concerns the assessment of histone protein abundance. In the present study, histone protein levels were evaluated by western blot analysis using β-actin as a loading control. Although this approach allowed comparison between experimental groups, normalization to a cytosolic protein may not fully account for variations in chromatin-associated proteins. Furthermore, the ModSpec® analysis performed in this study provided relative quantification of histone post-translational modifications and did not directly measure total histone abundance. Therefore, the observed changes in histone protein levels require further validation, and additional studies will be necessary to confirm alterations in histone abundance. Finally, the use of a systemic ID4-deficient mouse model at a single developmental stage represents another limitation of this study. The contribution of systemic or extra-hepatic factors to the observed phenotype cannot be excluded. Therefore, future studies using organ-specific ID4-deficient models at the mature stage, as well as direct functional metabolic assays, are important for clarifying the tissue-specific physiological functions of ID4 in energy metabolism.

Overall, our findings identify an association between ID4 deficiency, altered histone dynamics, chromatin accessibility, and impaired hepatic fatty acid synthesis. Rather than establishing a direct mechanistic pathway, the present study provides a framework for further investigation into how ID4 influences epigenomic regulation and metabolic homeostasis.

## 5 Conclusion

This study demonstrates that ID4 is associated with coordinated changes in chromatin accessibility, histone dynamics, and gene expression linked to hepatic lipid metabolism. These findings support a role for ID4 in epigenetic regulation of metabolic pathways, rather than acting solely through its conventionally proposed function as a transcriptional repressor. Overall, these results suggest that impaired hepatic lipid metabolism may contribute, at least in part, to the premature death observed in *Id4*^*−/−*^ mice; however, further studies are required to establish causal mechanisms.

## CRediT authorship contribution statement

**Yoshikazu Hayashi:** Writing – review & editing, Validation, Investigation, Formal analysis, Data curation. **Koji Kinoshita:** Writing – review & editing, Investigation, Formal analysis, Data curation. **Tsai-Ming Lu:** Writing – review & editing, Validation, Methodology, Investigation, Formal analysis, Data curation. **Hsin-Yi Tseng:** Writing – review & editing, Validation, Methodology, Investigation, Formal analysis, Data curation. **Keita Maki:** Validation, Investigation. **Soi Kimura:** Validation, Investigation. **Ena Yano:** Investigation. **Ayaka Saeki:** Investigation. **Atsushi Yasukochi:** Writing – review & editing. **Kento Minami:** Writing – review & editing. **Mayo Yamamura:** Writing – review & editing. **Ichiro Takahashi:** Writing – review & editing. **Masato Hirata:** Writing – review & editing. **Eijiro Jimi:** Writing – review & editing. **Lo Yi-Chen:** Writing – review & editing. **Cheng-Fu Kao:** Writing – review & editing, Methodology. **Tomoyo Kawakubo-Yasukochi:** Writing – review & editing, Writing – original draft, Validation, Supervision, Project administration, Methodology, Investigation, Funding acquisition, Formal analysis, Data curation, Conceptualization.

## Funding

This work was supported by the 10.13039/501100001691Japan Society for the Promotion of Science (10.13039/501100001691KAKENHI grants JP20H03854 and JP24K02868 to M.H.; JP22K09914, JP22KK0263, JP25K22690, and JP25K02802 to T.K.-Y.; JP23K09133 to Y·H.; JP22K17003 and JP24K19842 to E.Y.; JP23K19721 and JP25K20376 to S·K.; and JP22K10173 to A.Y.), 10.13039/100018289The Shinnihon Foundation of Advanced Medical Treatment Research, The 10.13039/100008731Nakatomi Foundation, 10.13039/100007449Takeda Science Foundation, 10.13039/501100007263Astellas Foundation for Research on Metabolic Disorders, Kakihara Science Technology Foundation, The Mochida Memorial Foundation for Medical and Pharmaceutical Research, Lotte Research Promotion Grant, Daiichi Sankyo Foundation of Life Science, Japan Diabetes Foundation, Novo Nordisk Pharma, and Mishima Kaiun Memorial Foundation.

## Declaration of competing interest

No competing financial interests exist.

## Data Availability

Data will be made available on request.
