# Supplementary material for: Inhibitor of DNA binding/differentiation 4 deficiency impairs hepatic fatty acid synthesis and is associated with epigenomic alterations in chromatin accessibility
Source: Mol Metab. 2026 Jul 8;111:102416. doi: 10.1016/j.molmet.2026.102416 (PMC13400251; doi:10.1016/j.molmet.2026.102416)
Supplement: Multimedia component 1 [file mmc1.docx]

**Supplemental Information:**

**Inhibitor of DNA binding/differentiation 4 is essential for hepatic fatty acid synthesis *via* epigenomic regulation of chromatin accessibility**

Yoshikazu Hayashi^a,b,c†^, Koji Kinoshita^a,d†^, Tsai-Ming Lu^e^, Hsin-Yi Tseng^e^, Keita Maki^a,d^, Soi Kimura^a^, Ena Yano^a^, Ayaka Saeki^a^, Atsushi Yasukochi^f^, Kento Minami^a^, Mayo Yamamura^g^, Ichiro Takahashi^d,h^, Masato Hirata^c^, Eijiro Jimi^a,g^, Lo Yi-Chen^i^, Cheng-Fu Kao^e^, Tomoyo Kawakubo-Yasukochi^a*^

^a^OBT Research Center, Faculty of Dental Science, Kyushu University, 3-1-1 Maidashi, Higashi-ku, Fukuoka 812-8582, Japan

^b^Division of Functional Structure, Department of Morphological Biology, Fukuoka Dental College, Tamura, Sawara-ku, Fukuoka 814-0193, Japan

^c^Oral Medical Research Center, Fukuoka Dental College, Tamura, Sawara-ku, Fukuoka 814-0193, Japan

^d^Section of Orthodontics and Dentofacial Orthopedics, Division of Oral Health, Growth and Development, Kyushu University Faculty of Dental Science, Fukuoka, Japan.

^e^Institute of Cellular and Organismic Biology, Academia Sinica, Taipei, Taiwan

^f^Section of Oral and Maxillofacial Oncology, Division of Maxillofacial Diagnostic and Surgical Sciences, Faculty of Dental Sciences, Kyushu University, 3-1-1 Maidashi, Higashi-ku, Fukuoka 812-8582, Japan

^g^Laboratory of Molecular and Cellular Biochemistry, Faculty of Dental Science, Kyushu University, 3-1-1 Maidashi, Higashi-ku, Fukuoka 812-8582, Japan

^h^Dental Maxillofacial Center, Kyushu University Hospital, 3-1-1 Maidashi, Higashi-ku, Fukuoka 812-8582, Japan

^i^Institute of Food Sciences and Technology, National Taiwan University, Taipei 10617, Taiwan

^†^These authors contributed equally to the work

^*^**Correspondence:**

Tomoyo Kawakubo-Yasukochi, DDS, PhD

OBT Research Center

Faculty of Dental Science

Kyushu University

3-1-1 Maidashi, Higashi-ku, Fukuoka 812-8582, Japan

Tel.: +81-92-642-6343

Email: [tomoyo@dent.kyushu-u.ac.jp](mailto:tomoyo@dent.kyushu-u.ac.jp)

***Supplementary Figure 1. Expression of histone acetylation-related enzymes and hepatic acetyl-CoA levels in the liver of Id4^+/+^ and Id4^−/−^ male mice at 3 weeks of age.***

(A–B) RT-qPCR analysis for HDACs (*Hdacs* 1, 2, 3, 4, 5, 6, 7, 8, 9, 10, and 11, and *Sirt1*, *Sirt6*, and *Sirt7*) (A) and HATs (*Gcn5*, *Hat1*, and *Cbp/P300*) (B). (C) Hepatic acetyl-CoA levels quantified by ion-pair LC-MS/MS (analysis performed by CERI; Chemicals Evaluation and Research Institute, Tokyo, Japan). Data are presented as mean ± SEM. Student’s t-test (two-tailed) was used. **P* < 0.05 and ***P* < 0.01.
