# Supplementary figures and images for: Inhibitor of DNA binding/differentiation 4 deficiency impairs hepatic fatty acid synthesis and is associated with epigenomic alterations in chromatin accessibility
Source: Mol Metab. 2026 Jul 8;111:102416. doi: 10.1016/j.molmet.2026.102416 (PMC13400251; doi:10.1016/j.molmet.2026.102416)

**A**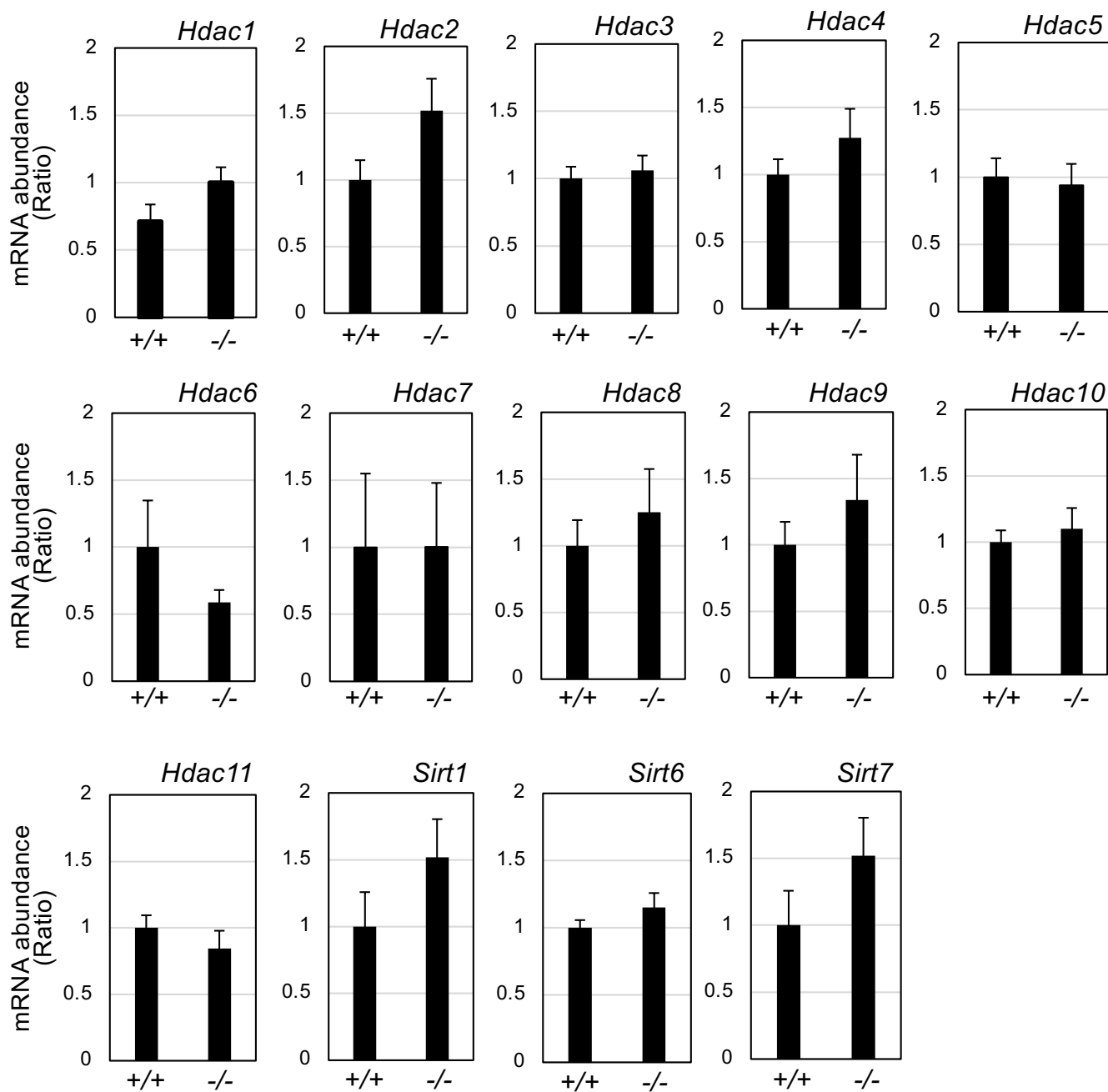**B**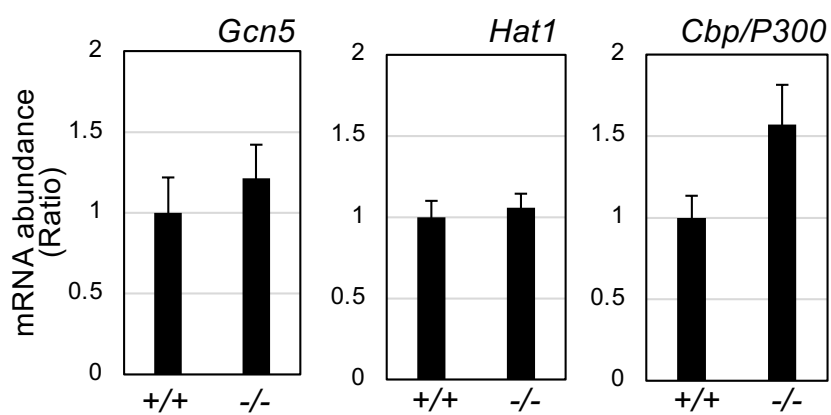Fig S1. Hayashi *et al.* (continued)

C

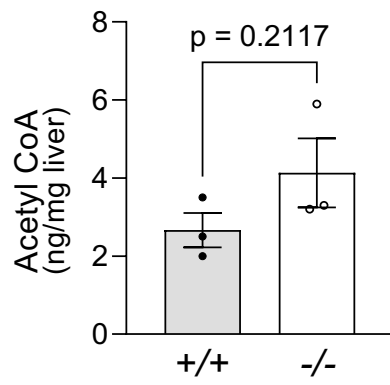

Supplement: Multimedia component 2 [file mmc2.pdf]
