# Supplementary material for: Inhibitor of DNA binding/differentiation 4 deficiency impairs hepatic fatty acid synthesis and is associated with epigenomic alterations in chromatin accessibility
Source: Mol Metab. 2026 Jul 8;111:102416. doi: 10.1016/j.molmet.2026.102416 (PMC13400251; doi:10.1016/j.molmet.2026.102416)
Supplement: Multimedia component 3 [file mmc3.docx]

***Supplementary Table 1. Primer sets for RT-qPCR analysis.***

**Gene Direction Primer sequence**

(mouse)

β-actin Forward GGC TGT ATT CCC CTC CAT CG

Reverse CCA GTT GGT AAC AAT GCC ATG

Acaca Forward ATG GGC GGA ATG GTC TCT TTC

Reverse TGG GGA CCT TGT CTT CAT CAT

Fasn Forward GGA GGT GGT GAT AGC CGG TAT

Reverse TGG GTA ATC CAT AGA GCC CAG

Srebf1 Forward GAT GTG CGA ACT GGA CAC AG

Reverse CAT AGG GGG CGT CAA ACA G

Atgl Forward CTG CCT GCC AGA CTC AAT G

Reverse GAA AGG GTG GTC ATC AGG TC

Hsl Forward CTT CCT GCA AGA GTA TGT CAC G

Reverse ATG GCA GGT GTG AAC TGG A

Elovl6 Forward CCC GAA CTA GGT GAC ACG AT

Reverse TAC TCA GCC TTC GTG GCT TT

Cd36 Forward GGA CAT TGA GAT TCT TTT CCT CTG

Reverse GCA AAG GCA TTG GCT GGA AGA AC

Pparg Forward GTA CTG TCG GTT TCA GAA GTG CC

Reverse ATC TCC GCC AAC AGC TTC TCC T

Adipoq Forward TGT TCC TCT TAA TCC TGC CCA

Reverse CCA ACC TGC ACA AGT TCC CTT
