# Supplementary material for: Inhibitor of DNA binding/differentiation 4 deficiency impairs hepatic fatty acid synthesis and is associated with epigenomic alterations in chromatin accessibility
Source: Mol Metab. 2026 Jul 8;111:102416. doi: 10.1016/j.molmet.2026.102416 (PMC13400251; doi:10.1016/j.molmet.2026.102416)
Supplement: Multimedia component 4 [file mmc4.docx]

***Supplementary Table 2. Primary antibodies for WB analysis.***

**Target Dilution Source and catalog number**

β-Actin 1:100000 MBL (Aichi, Japan), Cat# M177-3

Id4 1:500 Santa Cruz Biotechnologies (Dallas, TX, USA), Cat# sc365656

Acc1 1:60000 Proteintech (Rosemont, IL, USA), Cat# 67373

Fasn 1:100000 Abcam (Cambridge, UK), Cat# Ab128856

Atgl 1:3000 Proteintech, Cat# 55190

Hsl 1:3000 Cell Signaling Technology (Danvers, MA, USA), Cat# 18381

pHsl (S660) 1:3000 Cell Signaling Technology, Cat# 45804

Srebp1 1:500 Santa Cruz Biotechnologies, Cat# sc13551

Histone H1 1:500 Santa Cruz Biotechnologies, Cat# sc393358

Histone H2A 1:4000 Cell Signaling Technology, Cat# 12349

Histone H2B 1:4000 Cell Signaling Technology, Cat# 12364

Histone H3 1:4000 Cell Signaling Technology, Cat# 4499

Acetyl-Histone H3(K27) 1:4000 Cell Signaling Technology, Cat# 4353

Tri-Methyl-Histone H3(K4) 1:4000 Cell Signaling Technology, Cat# 9751

Histone H4 1:4000 Cell Signaling Technology, Cat# 13919

PPARγ 1:4000 Cell Signaling Technology, Cat# 2435

Adiponectin 1:4000 Cell Signaling Technology, Cat# 2789
